# Schlafen 11 as a prognostic and potentially predictive biomarker in soft tissue sarcoma: evidence from a real-world cohort

**DOI:** 10.3389/fonc.2026.1792367

**Published:** 2026-03-10

**Authors:** Adrian Georg Simon, Su Ir Lyu, David Stahl, Lars Mortimer Schiffmann, Birte Wenk, Reinhard Buettner, Nora Wuerdemann, Armin Tuchscherer, Roland Tillmann Ullrich, Alexander Quaas

**Affiliations:** 1 Institute of Pathology, University Hospital Cologne, Faculty of Medicine, University of Cologne, Cologne, Germany; 2 Department I of Internal Medicine/Centre for Integrated Oncology Aachen Bonn Cologne Duesseldorf, University Hospital Cologne, Faculty of Medicine, University of Cologne, Cologne, Germany; 3 Translational Research for Infectious Diseases and Oncology, University Hospital Cologne, Cologne, Germany; 4 Mildred Scheel School of Oncology Aachen Bonn Cologne Düsseldorf (MSSO ABCD), Cologne, Faculty of Medicine and University Hospital of Cologne, Cologne, Germany; 5 Department of General, Visceral, Thoracic and Transplant Surgery, University Hospital of Cologne, Faculty of Medicine, University of Cologne, Cologne, Germany; 6 Department for Orthopedics and Trauma Surgery, University Hospital Cologne, Faculty of Medicine, University of Cologne, Cologne, Germany

**Keywords:** soft tissue sarcoma, Schlafen 11, SLFN11, predictive biomarker, prognostic biomarker, trabectedin, DNA-damaging agents, precision medicine

## Abstract

**Background:**

Soft tissue sarcomas (STS) carry a high risk of relapse or metastasis even after complete resection. (Neo-)adjuvant chemotherapy benefits only a subset of patients, underscoring the need for predictive biomarkers. Schlafen 11 (SLFN11) has emerged as a marker of sensitivity to DNA-damaging agents. This study evaluated SLFN11 as a prognostic and predictive biomarker for (neo-)adjuvant chemotherapy in STS.

**Materials and methods:**

SLFN11 expression was assessed by immunohistochemistry in 242 patients with STS across different disease stages, using the H-score and percentage of positive tumor cells. Sub-cohorts included patients receiving neoadjuvant therapy (n = 33), primary resection (n = 193), palliative first-line chemotherapy (n = 26), or a palliative salvage therapy with trabectedin (n = 22). Associations between SLFN11 levels, clinicopathological features, and survival were analyzed.

**Results:**

In the neoadjuvant cohort, SLFN11 expression correlated with pathological tumor regression after chemotherapy alone (rho = 0.73, p = 0.016) and chemotherapy ± radiotherapy (rho = 0.62, p = 0.011). Among primarily resected STS treated with adjuvant chemotherapy ± radiotherapy, SLFN11-high tumors were associated with significantly longer overall survival (OS) (p = 0.007) and disease-free survival (DFS) (p = 0.022). SLFN11 was independently associated with improved outcome (OS: HR 0.06, p = 0.002; DFS: HR 0.08, p = 0.004). In the palliative first-line chemotherapy cohort, SLFN11-high tumors showed improved OS (p = 0.005) and progression-free survival (PFS) (p = 0.024), and SLFN11 remained independently predictive (OS: HR 0.11, p = 0.001). In the trabectedin cohort, SLFN11-high tumors demonstrated longer OS (p = 0.04) and PFS (p = 0.024).

**Conclusion:**

SLFN11 is a prognostic and potentially predictive biomarker in STS in the context of chemotherapy. Our results support a prospective validation, standardization of SLFN11 assessment, and consecutive clinical implementation.

## Introduction

Soft tissue sarcomas (STS) are a heterogeneous group of cancers comprising more than 70 distinct entities, with undifferentiated pleomorphic sarcoma (UPS), dedifferentiated liposarcoma (ddLPS), and leiomyosarcoma (LMS) being the most common subtypes (1). Surgical resection, with or without perioperative chemotherapy and/or radiotherapy, remains the primary curative treatment for localized STS (2). However, 20-30% of patients experience local recurrence, and up to 40% develop distant metastases (3, 4). In advanced, unresectable, or metastatic STS, therapeutic options are limited to radiotherapy and/or chemotherapy, with modest overall response rates up to 35%, most commonly involving anthracyclines such as doxorubicin alone or in combination with DNA-damaging agents including ifosfamide or dacarbazine (2, 5). As a second- or later-line treatment, trabectedin, an alkylating agent that interferes with DNA repair, is commonly used in metastatic high-grade ddLPS, LMS, or more uncommon sarcomas (2, 6–8). Both first-line chemotherapy and trabectedin are associated with high rates of grade 3–4 toxicity, affecting up to 70-80% of patients, with neutropenia, anemia, febrile neutropenia, and transaminase elevations among the most common adverse events (5, 8, 9). Despite all therapeutic efforts, the prognosis of metastatic STS remains poor, with 5-year overall survival rates generally below 20% (10, 11). Given the limited efficacy of current systemic therapies and the substantial biological heterogeneity of STS, there is a critical need for predictive biomarkers that can identify patients most likely to benefit from specific DNA-damaging treatments. One such emerging biomarker is Schlafen family member 11 (SLFN11). SLFN11 has recently emerged as a central regulator of cellular response to DNA stress and is recruited to single-strand DNA damage sites via the Replication Protein A (RPA) complex (12). Upon binding to the DNA damage sites, SLFN11 blocks replication-fork progression, leading to fork collapse (12, 13). Additionally, SLFN11 disrupts ATR–CHK1 checkpoint signaling and impairs DNA-repair processes, which further drives cells toward apoptosis when replication stress persists (12, 14).

Because of these key functions in DNA damage repair, SLFN11 has recently emerged as a potential predictive biomarker for response to DNA-damaging agents, including platinum-based compounds, anthracyclines such as doxorubicin, alkylating agents such as trabectedin, topoisomerase inhibitors, and PARP inhibitors (15, 16). Retrospective studies have reported improved response rates and survival in several cancers with high SLFN11 expression treated with DNA-damaging agents, including various carcinomas (16–21), and pediatric sarcomas, mostly including Ewing sarcomas (22–24). To date, however, no study has explored the potential of SLFN11 as a predictive or prognostic biomarker in adult soft tissue sarcomas. Therefore, in this study, we investigate SLFN11 protein expression as a potential prognostic and predictive biomarker in a comprehensive STS cohort across multiple treatment stages.

## Materials and methods

### Soft tissue sarcoma cohort and sub-cohorts of the University Hospital Cologne

**Figure 1:**
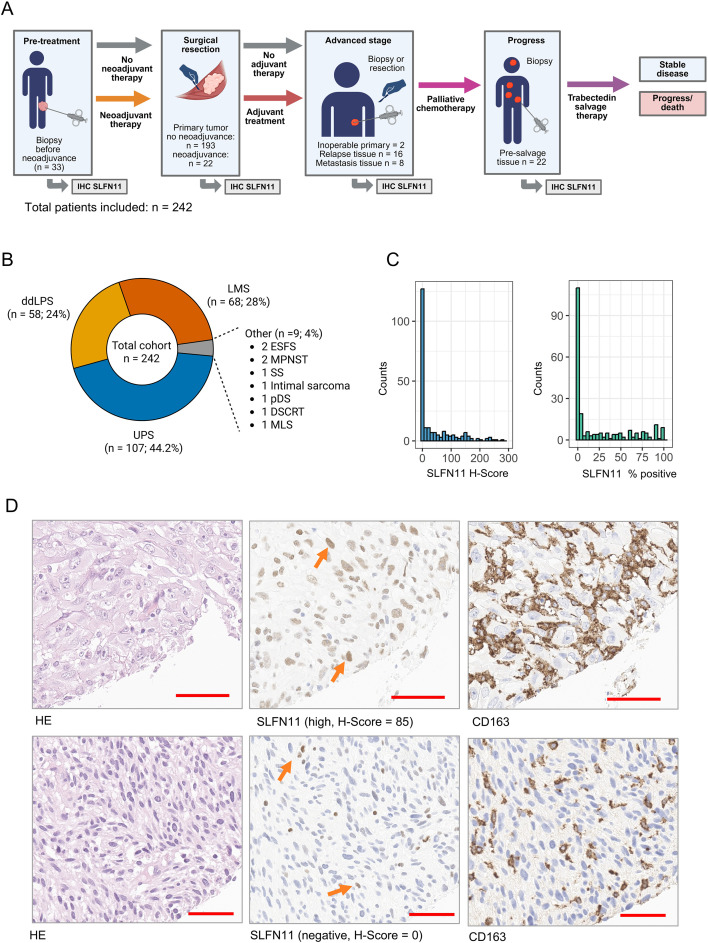
STS cohort, study workflow, and SLFN11 expression characteristics. **(A)** Schematic overview of workflow, tissue sampling, and SLFN11 assessment strategy. SLFN11 immunohistochemistry (IHC) was performed on samples obtained at different clinical stages, including pre-treatment biopsies, primary tumor resections (with or without neoadjuvant therapy), and advanced-stage, relapse, and/or metastatic biopsies Created in BioRender. Simon, A. G. (2026) https://BioRender.com/s1gfg0p. **(B)** Distribution of histological STS subtypes. **(C)** Distribution of SLFN11 expression: H-score (left) and SLFN11-positive tumor cell percentage score (right) across the entire cohort. Both metrics show a right-skewed distribution with a substantial number of SLFN11-negative or low-expressing cases. **(D)** Representative examples of high and absent SLFN11 expression in tumor cells. *Top row:* Tumor with high SLFN11 expression (H-Score = 230), showing strong nuclear staining and a diffuse inflammatory infiltrate (orange arrows). CD163 immunohistochemistry highlights tumor-associated macrophages within inflammatory regions, which were excluded from SLFN11 scoring based on morphology and CD163 positivity. *Bottom row:* Tumor with absent SLFN11 expression (H-Score = 0), with only scattered inflammatory cells showing SLFN11 positivity (orange arrows), confirmed as macrophages by CD163 staining and not included in tumor cell scoring. Red bar = 50 µm. IHC, immunohistochemistry; STS, soft tissue sarcomas; ddLPS, dedifferentiated liposarcoma; DSRCT, desmoplastic small round cell tumor; ESFS, epithelioid sclerosing fibrosarcoma; LMS, leiomyosarcoma; MLS, myxoid liposarcoma; MPNST, malignant peripheral nerve sheath tumor; pDS, pleomorphic dermal sarcoma; SS, synovial sarcoma; UPS, undifferentiated pleomorphic sarcoma.

All patients were treated at the surgical departments of the University Hospital Cologne (depending on anatomical tumor site and surgical procedure, see affiliations) and/or the Department I of Internal Medicine/Oncology of the University Hospital Cologne between 2013 and 2025. A total of n = 242 patients with soft tissue sarcomas (STS) in various stages of disease were included (**Table 1**, **Figure 1A**). Sub-cohorts of patients receiving neoadjuvant treatment (**Table 1**, **Supplementary Table 1**), palliative systemic chemotherapy in an advanced, relapsed and/or metastatic setting (**Table 1**, **Supplementary Table 2**), and trabectedin in a palliative salvage treatment regimen (**Table 1**, **Supplementary Table 3**) were formed (details: see Results section).

**Table 1:** University Hospital Cologne: Soft tissue sarcoma cohort and sub-cohorts.

Variable		All soft tissue sarcomas (n = 242)	Neoadjuvant cohort (n = 33)	Primary surgery cohort (n = 193)	Palliative chemotherapy cohort (n = 26)	Palliative salvage cohort: Trabectidin (n = 22)
		n (%)	n (%)	n (%)	n (%)	n (%)
Tissue						
Primary tissue		-*	33 (100)	193 (100)	2 (8)	3 (14)
Local relapse		-*	0 (0)	0 (0)	16 (62)	14 (64)
Distant Metastasis		-*	0 (0)	0 (0)	8 (31)	5 (23)
Histology						
UPS		107 (44)	22 (67)	82 (43)	10 (38)	6 (27)
ddLPS		58 (24)	8 (24)	45 (23)	9 (35)	6 (27)
LMS		68 (28)	2 (6)	61 (32)	5 (19)	4 (18)
Other**		9 (4)	1 (3)	5 (3)	2 (8)	6 (27)
Sex						
Male		129 (53)	20 (61)	101 (52)	16 (62)	11 (50)
Female		113 (47)	13 (39)	92 (48)	10 (38)	11 (50)
Primary site						
Extremity		83 (34)	20 (61)	61 (32)	7 (27)	4 (18)
Retroperitoneum		57 (24)	6 (18)	47 (24)	6 (23)	4 (18)
Trunk		20 (8)	3 (9)	17 (9)	2 (8)	1 (5)
Abdominopelvic cavity		20 (8)	0 (0)	14 (7)	5 (19)	6 (27)
Visceral		36 (15)	1 (3)	32 (17)	4 (15)	4 (18)
Thoracic cavity		8 (3)	2 (6)	6 (3)	2 (8)	2 (9)
Head and neck		11 (5)	0 (0)	10 (5)	0 (0)	1 (5)
Other		7 (3)	1 (3)	6 (3)	0 (0)	0 (0)
R status						
R0		133 (55)	18 (55)	111 (58)	11 (42)	10 (45)
R1		61 (25)	6 (18)	50 (26)	9 (35)	6 (27)
Rx		48 (20)	8 (24)	32 (17)	6 (23)	6 (27)
FNCLCC***						
1		14 (6)	1 (3)	12 (6)	1 (4)	0 (0)
2		70 (29)	5 (15)	61 (32)	4 (15)	6 (27)
3		155 (64)	27 (82)	118 (62)	21 (81)	14 (64)
NA		3 (1)	0 (0)	2 (1)	0 (0)	2 (9)
Neoadjuvant therapy						
chemotherapy		13 (5)	13 (39)	0 (0)	0 (0)	0 (0)
radiation		15 (6)	14 (42)	0 (0)	2 (8)	3 (14)
radiochemotherapy		6 (2)	6 (18)	0 (0)	1 (4)	0 (0)
no neoadjuvant treatment		200 (83)	0 (0)	193 (100)	23 (88)	16 (73)
NA		8 (3)	0 (0)	0 (0)	6 (23)	3 (14)
Adjuvant therapy						
chemotherapy		26 (11)	3 (9)	22 (11)	2 (8)	6 (27)
radiation		72 (30)	8 (24)	58 (30)	7 (27)	4 (18)
radiochemotherapy		8 (3)	0 (0)	8 (4)	1 (4)	2 (9)
no adjuvant treatment		115 (48)	20 (61)	91 (47)	9 (35)	7 (32)
NA		24 (10)	2 (6)	14 (7)	7 (27)	3 (14)

*The complete sarcoma cohort contains various subgroups, with some patients being part of several sub-cohorts, from which primary, relapsed and metastatic tissue at various points during disease was obtained. Thus, the sub-cohorts add up to a larger number than the total cohort.

**Includes 2 malignant peripheral nerve sheath tumors (MPNST), 2 epithelial sclerosing fibrosarcomas (ESFS), 1 intima sarcoma, 1 synovial sarcoma, 1 myxoid liposarcoma, 1 pleomorphic dermal sarcoma, 1 desmoplastic small-round cell tumor (DSRCT).

***Grading according to the Fédération Nationale des Centres de Lutte Contre le Cancer, including tumor differentiation, tumor necrosis, and mitoses per 10 HPF/2 mm^2^. NA = Not assessed for ESFS, DSRCT.

NA not available/not assessed.

### Generation of tissue microarrays and immunohistochemical staining

Tissue samples were assessed by a trained soft tissue pathologist (AGS) for feasibility. For the assessment of SLFN11 protein expression, tissue microarrays (TMAs) were generated as reported before (25, 26): Four tissue cores (diameter 1.1 mm) from representative tumor areas were transferred to a recipient paraffin block using a manual precision instrument. Necrotic areas were avoided. Benign tonsil tissue and appendix tissue were used as controls. Additionally, we conducted whole-section staining for n = 50 tumors, including biopsies and large tissue sections for assessment of SLFN11 distribution. Immunohistochemical staining (IHC) for SLFN11 was conducted using an anti-human SLFN11 Rabbit monoclonal antibody (Cell Signaling, clone D8W1B, RRID: AB_2799063; dilution: 1:100; pre-treatment: EDTA at 100°C, 20 min; peroxidase block 5 min; primary antibody 20 min, secondary antibody 8 min) on a Leica Bond Max platform (RRID: SCR_026887; Leica, Wetzlar, Germany) with a Polymer Refine Detection Kit (8 min) (RRID: AB_2891238; Leica). For macrophages, immunohistochemical staining for CD163 (Antibody: Cell Marque Monoclonal Mouse Anti-Human CD163, Clone MRQ-26, RRID: AB_1159119; dilution 1:100) was performed on a Leica Bond Max platform according to routine protocols at the Institute of Pathology of the University Hospital Cologne.

### Assessment of SLFN11 expression

Immunohistochemical SLFN11 protein expression was assessed using the semi-quantitative H-score method as reported before (25): Each TMA core was independently assessed by two trained pathologists (AGS, SIL). Staining intensities (negative = 0, weak = 1, moderate = 2, strong = 3) were multiplied for each core by the percentages of stained cells for each intensity fraction. These fractions were then added to an H-score (minimum: 0, maximum: 300). The median H-score of all four cores per tumor sample was used for further statistical analyses. Additionally, a percentage score was generated, estimating the percentage of all positive tumor cells of any intensity. SLFN11 expression was evaluated exclusively in tumor cell nuclei. Cells with morphological features consistent with macrophages (abundant cytoplasm, ovoid or kidney-shaped nuclei without atypia, broad cytoplasmic rim) were excluded from scoring. To support accurate discrimination between tumor cells and macrophages, CD163 IHC was performed on representative sections, and macrophage-rich areas were identified and excluded from SLFN11 scoring. Discordant cases were discussed on a multi-head microscope and consensus was reached. To assess inter-observer variability, the Intraclass Correlation Coefficient (ICC; Two-way model, type: agreement) was used.

### Data preparation, visualization, and statistical analysis

All data processing, visualization, and statistical analyses were conducted with R (v4.2.3) and RStudio (v2024.12.1). Interdependencies between clinical data, histopathological data, and SLFN11 expression were evaluated using appropriate statistical tests, with an adjusted p-value ≤ 0.05 considered significant, indicated in the results section.

Overall survival (OS) was calculated from the date of diagnosis (or, if not available, date of surgery) until the date of death (from any cause) or loss to follow-up. Disease-free survival (DFS) was calculated from the end of (first) treatment to the date of first relapse, first metachronous metastasis, or death (from any cause). Progression-free survival (PFS) was calculated from the start of progression-specific therapy to progression in radiological imaging, as assessed by a consultant radiologist, death, or censored (patient alive with stable disease). Progression was defined as a clinically relevant tumor growth according to RECIST criteria (27) detected in imaging (CT or MRI) and/or new metastasis. For survival analyses, Kaplan-Meier curves, a log-rank test, univariate analyses, and a multivariable Cox proportional hazards model were used, with a p-value ≤ 0.05 considered significant and a p-value ≤ 0.1 considered a statistical trend.

### Use of large language models

The authors used ChatGPT (OpenAI, GPT-5 model) to assist with grammar, spelling, and readability improvement in preparing this manuscript. The scientific content and scientific conclusions are solely the responsibility of the authors.

## Results

### SLFN11 expression across soft tissue sarcoma subtypes

The complete cohort comprised 242 STS patients across major histologic subtypes including undifferentiated pleomorphic sarcoma (UPS), leiomyosarcoma (LMS), dedifferentiated liposarcoma (ddLPS), and nine less common sarcomas (**Table 1**, **Figure 1B**). Because both the H-score method and assessment of overall percentage tumor cells have been used across different cancers (17, 22, 23, 28, 29), we used both metrics in our initial assessment. Overall nuclear SLFN11 expression levels were low across the whole cohort (baseline expression assessed in n = 222 untreated STS samples; global median H-score and percentage score: 2.5; ranges 0–280 and 0-100%) (**Figure 1C**), with ddLPS showing the highest SLFN11 expression (median H-score and percentage score: 10) and LMS the lowest expression (median H-score and percentage score: 0). 118/222 tumors (52%) showed at least a focal positivity for SLFN11 (H-score ≥ 1; ≥ 1% positive tumor cells). SLFN11 expression was consistent in whole-tissue sections, but showed increased accumulation in heavily inflamed areas: In these sections, CD163 staining confirmed abundant infiltration of often SLFN11-positive macrophages, which facilitated distinction from tumor cells especially in more monotonous, spindle-shaped tumors (**Figure 1D**). Interobserver agreement was good for the H-score (ICC(A,1) = 0.75; 95% CI: 0.67–0.82; p < 0.001) and excellent for the percentage score (ICC(A,1) = 0.86; 95% CI: 0.81–0.90; p < 0.001), indicating highly consistent SLFN11 assessment across both methods. Dichotomization by global SLFN11 median values resulted in identical groupings for the H-score and percentage-based method (both medians = 2.5/2.5%). Consequently, the H-score was used for subsequent analyses.

### SLFN11 correlates with tumor regression after neoadjuvant chemotherapy

**Figure 2:**
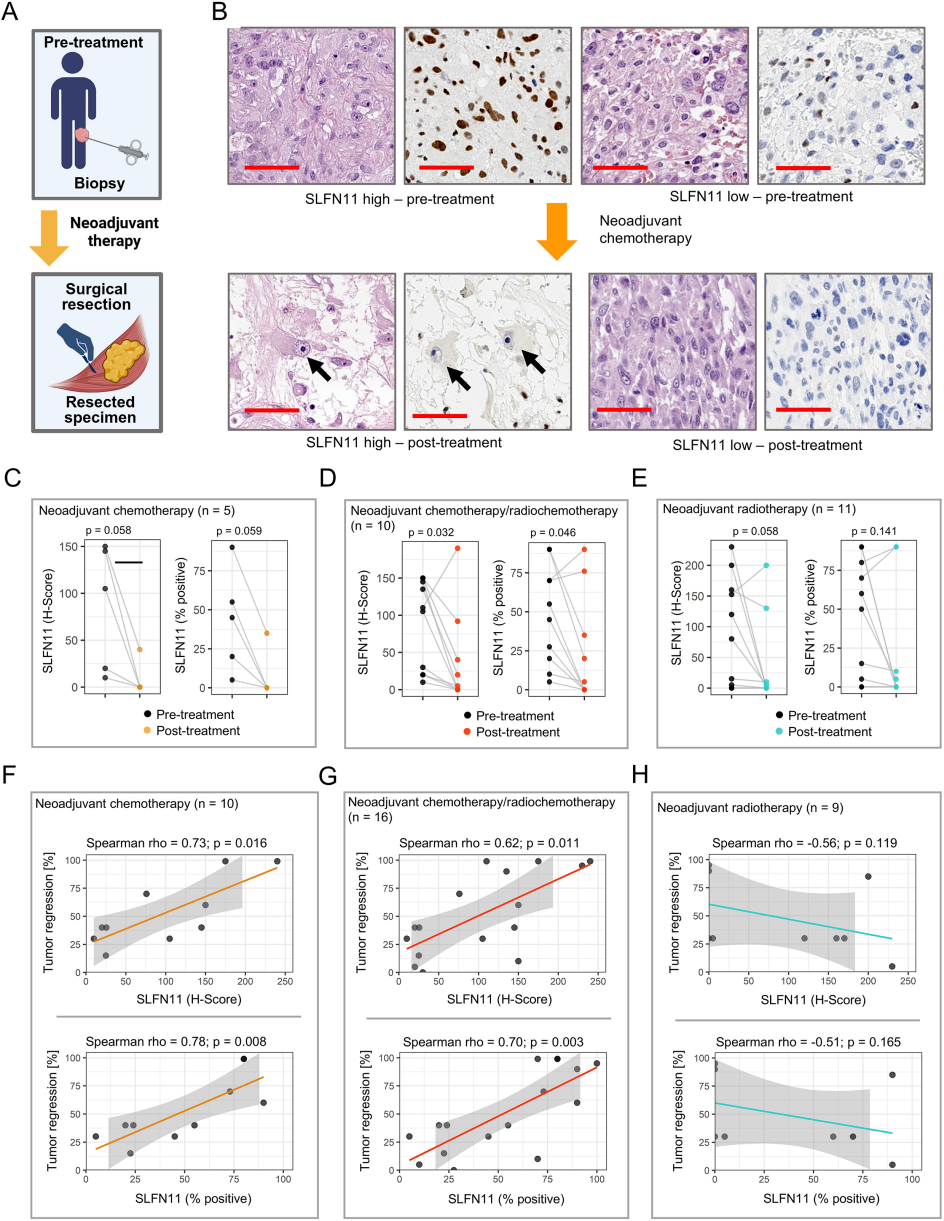
SLFN11 expression in sub-cohort treated with neoadjuvant chemotherapy. **(A)** Sub-cohort study design. Paired pre-treatment tumor biopsies and surgically resected specimens obtained after neoadjuvant chemotherapy were analyzed for SLFN11 expression by immunohistochemistry (IHC) (Paired samples available: n = 21) Created in BioRender. Simon, A. G. (2026) https://BioRender.com/aylpnnf. **(B)** Representative H&E and SLFN11 IHC images illustrating tumors with high or low SLFN11 expression before neoadjuvant chemotherapy and the corresponding post-therapy samples. Arrows indicate residual SLFN11-negative tumor cells after treatment; scale bars = 50 µm. **(C–E)** Paired analyses of SLFN11 expression in individual patients (n, 21) before and after neoadjuvant chemotherapy alone **(C)**, chemotherapy and radiochemotherapy **(D)**, and radiotherapy alone **(E)**, quantified as H-score and as percentage score of SLFN11-positive tumor cells. P values by paired Wilcoxon signed-rank test. **(F–H)** SLFN11 expression in untreated biopsy tissue before neoadjuvant chemotherapy correlates positively with tumor regression (in %) after neoadjuvant chemotherapy **(F)** and radiochemotherapy **(G)**, but not after neoadjuvant radiation **(H)**; Rho and p values assessed by Spearman’s rank correlation. Shaded areas denote 95% confidence intervals.

We generated a sub-cohort of 33/242 patients (14%) who received neoadjuvant therapy and for whom untreated biopsy tissue prior to neoadjuvant treatment was available (**Figure 2A**, **Supplementary Table 1**). Of these, 10/33 patients (30%) received chemotherapy alone, 6/33 (18%) combined radiochemotherapy, 3/33 (9%) chemotherapy combined with regional hyperthermia, and 14/33 patients (42%) neoadjuvant radiotherapy. Most patients treated with chemotherapy, with or without radiation, received a doxorubicin-based regimen, typically combined with ifosfamide (13/16; 71%) (**Supplementary Table 1**). Radiotherapy typically consisted of a total dose of 50.0 Gy delivered in fractions ranging from 1.8 Gy to 3.0 Gy.

Matched pre- and post-treatment specimens were available for 21/33 patients. In exploratory analyses, we observed a trend toward reduced post-treatment SLFN11 expression in both H-score (p = 0.059) and percentage score of positive cells (p = 0.058) in n = 5 patients treated with neoadjuvant chemotherapy alone (**Figures 2B, C**). When matched samples of patients treated with chemotherapy and radiochemotherapy were combined (n = 10), SLFN11 expression was significantly lower after treatment (H-score: p = 0.032; percentage score: p = 0.046) (**Figure 2D**). Patients treated with radiation alone (n = 11) also showed lower SLFN11 expression, although the difference did not reach significance (H-score: p = 0.058; percentage score: p = 0.141) (**Figure 2E**).

To explore whether SLFN11 expression correlated with pathological tumor regression, we analyzed all patients with available pathological regression data (in %) (including patients without paired samples). Despite small sample sizes, in the chemotherapy-only sub-group (n = 10), higher SLFN11 expression in pre-treatment biopsies strongly correlated with greater tumor regression after neoadjuvant chemotherapy (H-score: rho = 0.73, p = 0.016; percentage score: rho = 0.78, p = 0.008) (**Figure 2F**). A similar association was observed in patients receiving chemotherapy with radiation (n = 16) (H-score: rho = 0.62, p = 0.011; percentage score: rho = 0.70, p = 0.003) (**Figure 2G**). In contrast, no significant correlation was seen in patients treated with radiation alone (n = 9) (**Figure 2H**).

SLFN11 expression was not associated with overall survival (OS) or disease-free survival (DFS) in any treatment group (all log-rank p > 0.05), although the limited sample sizes restrict definitive conclusions.

### SLFN11 expression is associated with favorable outcome after adjuvant chemotherapy

We assessed SLFN11 expression in 193 primarily resected STS (**Table 1**). 31 patients (16%) received adjuvant chemotherapy or radiochemotherapy, 62 (32%) received adjuvant radiotherapy alone, and 89 (46%) received no adjuvant treatment; in 10 patients (5%), post-surgical follow-up was unavailable due to referral to external treatment centers.

Consistent with baseline SLFN11 distribution in primary tissue, higher SLFN11 expression was more frequently observed in ddLPS (SLFN11-high vs. -low: n = 26 vs. 19; 58% vs. 42%), whereas lower SLFN11 expression was more common in LMS (SLFN11-low vs. -high: n = 41 vs. 20; 67% vs. 33%). UPS cases were approximately equally distributed (SLFN11-low vs. -high: n = 40 vs. 42; 48% vs. 52%) (Fisher’s exact test, p = 0.045). SLFN11-high tumors were also more frequently incompletely resected (R1) compared with SLFN11-low tumors (41% vs. 24%; p = 0.037). However, none of these associations remained significant after Bonferroni correction for multiple comparisons (**Supplementary Table 4**). Follow-up data for OS were available for 190/193 samples (98%), and DFS data for 179/193 patients (93%).

**Figure 3:**
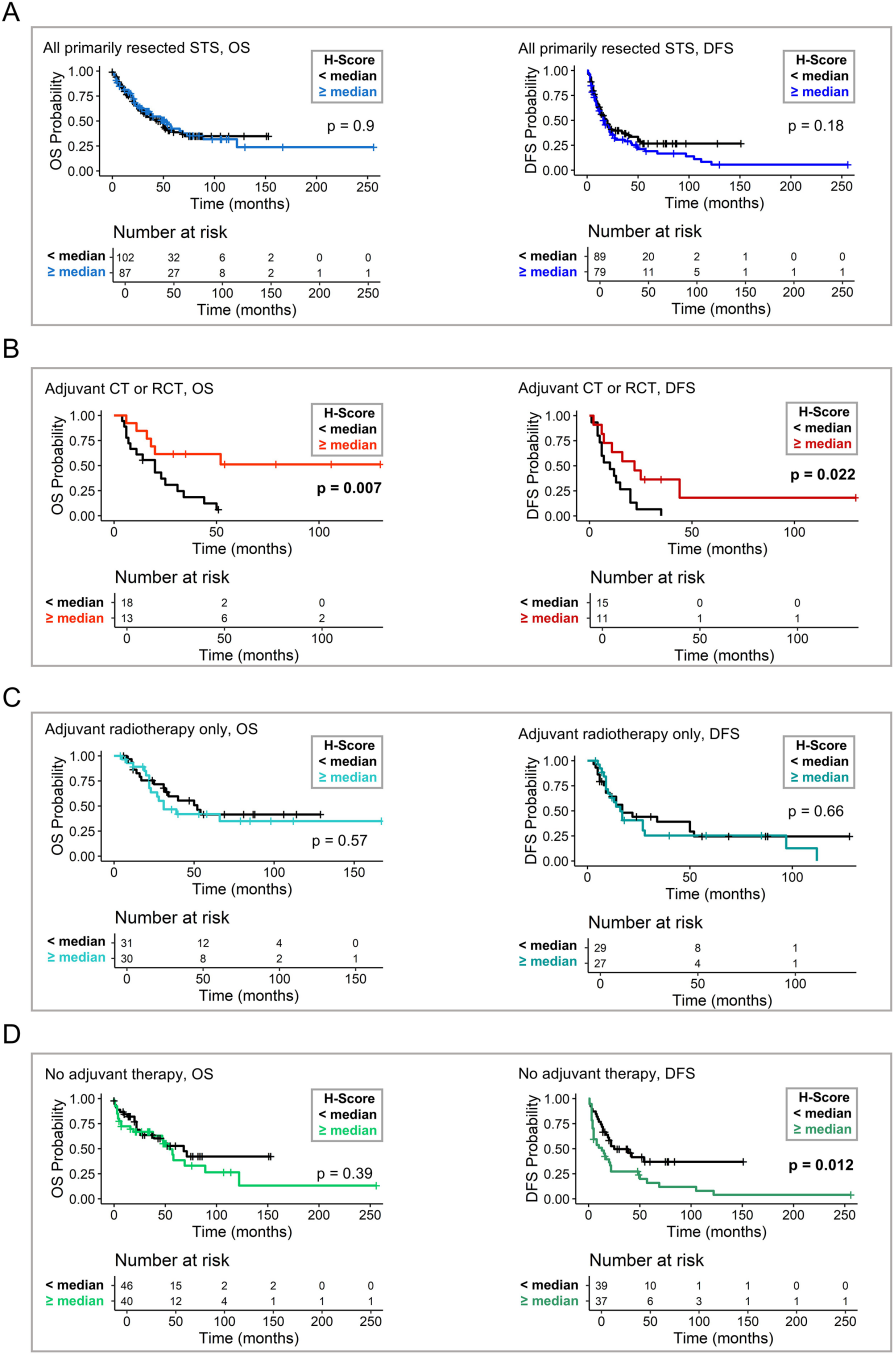
SLFN11 expression and survival in sub-cohort of primarily resected sarcomas. **(A)** Kaplan–Meier curves for overall survival (OS) and disease-free survival (DFS) in the sub-cohort of primarily resected soft tissue sarcoma, stratified by median SLFN11 expression (H-score). **(B)** Association of SLFN11 expression and OS/DFS in patients receiving adjuvant chemotherapy or chemoradiotherapy. **(C)** Association of SLFN11 expression and OS/DFS in patients receiving adjuvant radiotherapy only. **(D)** Association of SLFN11 expression and OS/DFS in patients receiving no adjuvant therapy after surgical resection. For all panels, survival differences were assessed using the log-rank test. Significant p values (p ≤ 0.05) are indicated in bold. STS, soft tissue sarcomas; CT, chemotherapy; RCT, radiochemotherapy.

In the overall STS cohort, SLFN11 expression was not associated with OS or DFS (**Figure 3A**). In contrast, among patients receiving adjuvant chemotherapy or radiochemotherapy (**Supplementary Table 5**), high SLFN11 expression correlated with significantly longer OS (35 vs. 17 months, p = 0.007) and longer DFS (22 vs. 10 months, log-rank p = 0.022) (**Figure 3B**). In univariate analyses, SLFN11 expression was significantly associated with OS (HR 0.28, p = 0.013) and DFS (HR 0.35, p = 0.026) (**Supplementary Table 6**). For multivariable Cox regression analyses, given the limited number of events in this subgroup, we restricted the model to the clinically most relevant covariables to avoid overfitting, including age at diagnosis, FNCLCC grade and R status (surgical margin status). SLFN11 remained an independent predictor of improved OS (HR 0.06, p = 0.002) and DFS (HR 0.08, p = 0.004) (**Supplementary Table 7**). Together, these analyses identify SLFN11 as an independent prognostic factor for OS and DFS in patients treated with adjuvant chemotherapy ± radiotherapy. To examine whether the prognostic effect of SLFN11 differed by treatment modality, we fitted a Cox model including an interaction between SLFN11 status and treatment type. In patients receiving adjuvant chemotherapy alone (n = 23), high SLFN11 expression was associated with a reduced hazard of death (HR 0.42, 95% CI 0.13–1.30), although not statistically significant. The association appeared stronger in the radiochemotherapy subgroup (n = 8; HR 0.11, 95% CI 0.01–1.05), and the interaction term suggested a potential differential benefit (p = 0.10). While exploratory given the small subgroup sizes, these findings indicate that SLFN11 may be particularly relevant in patients receiving both adjuvant chemotherapy and radiotherapy.

Notably, in patients receiving only adjuvant radiotherapy, no significant association between SLFN11 status and OS or DFS was observed (**Figure 3C**). In patients who received no adjuvant therapy, SLFN11 expression was not associated with OS, but with reduced DFS (13 vs. 20 months, p = 0.012) (**Figure 3D**). High levels of SLFN11 expression were associated with shorter DFS in univariate analyses (HR 2.01, p = 0.013) and multivariable analyses, including age, sex, FNCLCC grade and R status as covariables (HR 2.1, p = 0.030) (**Supplementary Table 8**).

### SLFN11 expression varies across course of disease

**Figure 4:**
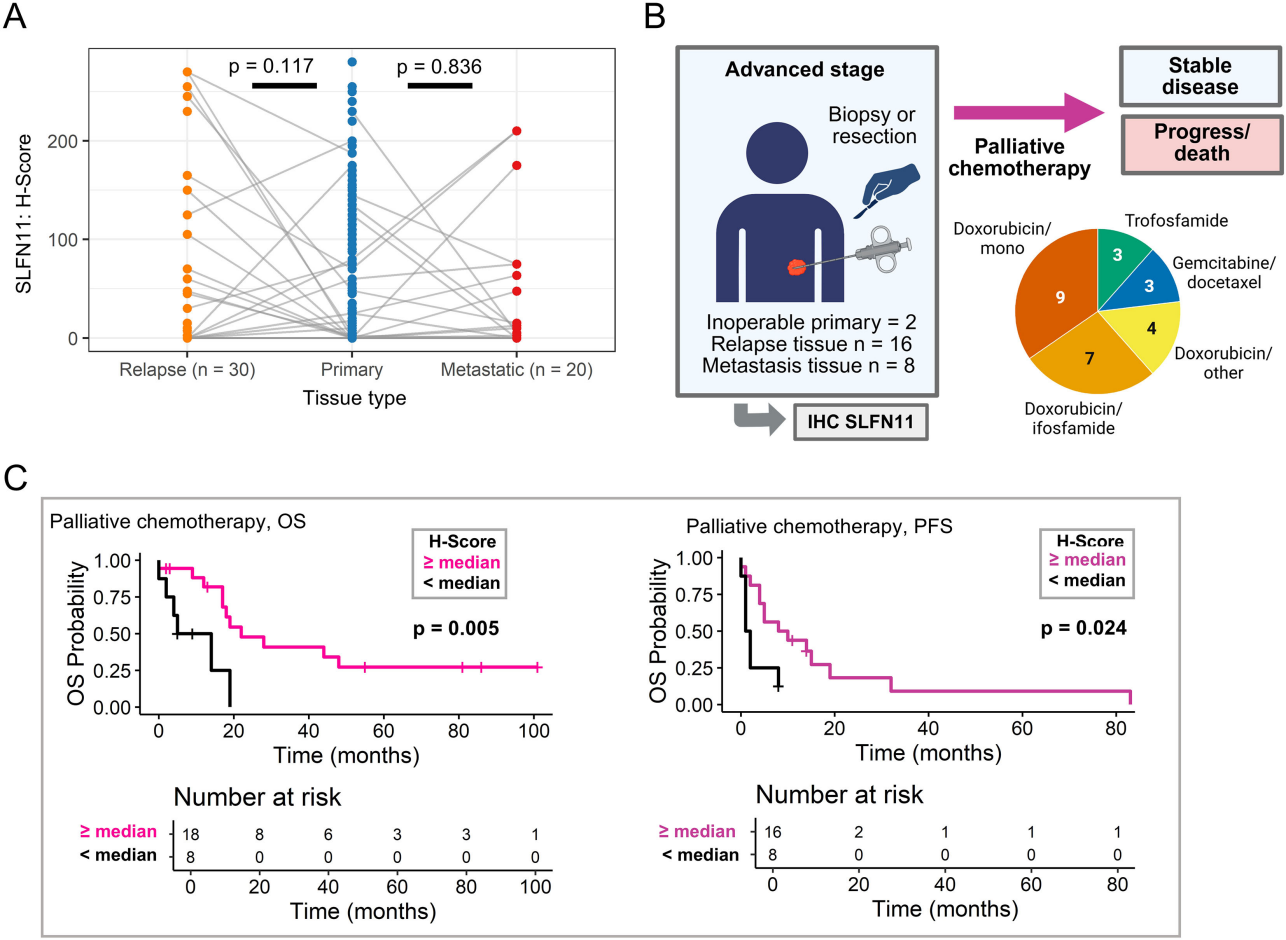
SLFN11 expression and survival in sub-cohort of patients treated with palliative chemotherapy. **(A)** Expression of SLFN11 in primary tumor tissue (central), local relapse (left) and distant metastasis (right). Grey lines indicate patient matched samples. P-values indicate results of a paired Wilcoxon test between primary tissue and relapsed tissue or metastatic tissue. **(B)** Sub-cohort of patients with unesectable, relapsed, or metastatic STS treated with palliative chemotherapy Created in BioRender. Simon, A. G. (2026) https://BioRender.com/cqyc5u0. **(C)** Association of SLFN11 expression and overall survival (OS)/progression-free survival (PFS) in patients receiving palliative chemotherapy. Survival differences were assessed using the log-rank test. Significant p values (p ≤ 0.05) are indicated in bold. STS, soft tissue sarcomas; IHC, immunohistochemistry.

We next assessed whether SLFN11 expression differed among primary, local relapse, and distant metastatic tissues. Metastatic tissue samples (n = 21; 13 UPS, 5 LMS, 2 ddLPS, 1 pleomorphic dermal sarcoma) showed higher median SLFN11 expression than primary tumors (median H-score and percentage score: both 10), whereas relapse samples (n = 42; 19 UPS, 10 LMS, 13 ddLPS) exhibited expression levels comparable to primary tissue (median for both: 1.25). Matched primary–relapse pairs were available for 30 patients, and primary–metastatic pairs for 20 patients. Although differences between SLFN11 expression in matched relapsed and metastatic tissue compared to primary tissue were common, these differences were not statistically significant (paired Wilcoxon test, all p ≥ 0.05) (**Figure 4A**). We thus used the global SLFN11 median (H-score and percentage score both 2.5) for further dichotomization in the palliative chemotherapy cohort and the trabectedin cohort as well (see below).

### SLFN11 expression predicts longer survival in palliative chemotherapy

Given the association of SLFN11 with outcomes in the adjuvant setting, we next examined whether SLFN11 also predicted benefit from systemic therapy in advanced disease. We generated a sub-cohort of 26 patients with unresectable primary tumors (n = 2), locally advanced irresectable relapse (n = 16), or distant metastases (n = 8) who received palliative systemic chemotherapy, and performed exploratory SLFN11 assessment (**Figure 4B**). The sub-cohort comprised 10 UPS, 9 ddLPS, 5 LMS, one intimal sarcoma, and one MPNST (**Table 1**, **Supplementary Table 2**). Most patients in the palliative chemotherapy cohort had previously undergone surgical resection, and prior (neo-)adjuvant chemotherapy and/or radiotherapy were documented where applicable (**Supplementary Table 2**). The majority received palliative systemic therapy as first-line treatment for advanced disease (20/26; 76%), whereas 6/26 (24%) received later-line treatment. SLFN11 expression was assessed in the last available tumor specimen obtained prior to initiation of palliative therapy. Distant metastases most often involved the lungs, brain, and bone. Most patients (20/26; 76%) received doxorubicin-based chemotherapy, frequently in combination with ifosfamide or docetaxel (**Supplementary Table 2**). Three patients (3/26; 12%) received gemcitabine plus docetaxel, and three (3/26; 12%) received trofosfamide (**Figure 4B**). We assessed SLFN11 expression in the last available tissue sample obtained prior to initiation of chemotherapy and dichotomized patients using the global SLFN11 median (H-score 2.5 and percentage score: 2.5%). Patients with high SLFN11 expression had a significantly longer OS (defined from start of palliative treatment to death of any cause) (18.5 vs. 5 months, log-rank p = 0.005) and a significantly longer progression-free survival (PFS) (10 vs. 1.5 months, log-rank p = 0.024) (**Figure 4C**). In univariate analyses high SLFN11 expression was associated with longer OS (HR 0.22, p = 0.009) and PFS (HR 0.31, p = 0.027) (**Supplementary Table 9**) and remained an independent predictor in multivariable analyses both for longer OS (HR 0.11, p = 0.001) and PFS (HR 0.18, p = 0.009), including age at treatment start and tissue subtype (metastatic vs. non-metastatic) as covariables (**Supplementary Table 10**).

### SLFN11 is associated with prolonged survival in palliative trabectedin treatment cohort

**Figure 5:**
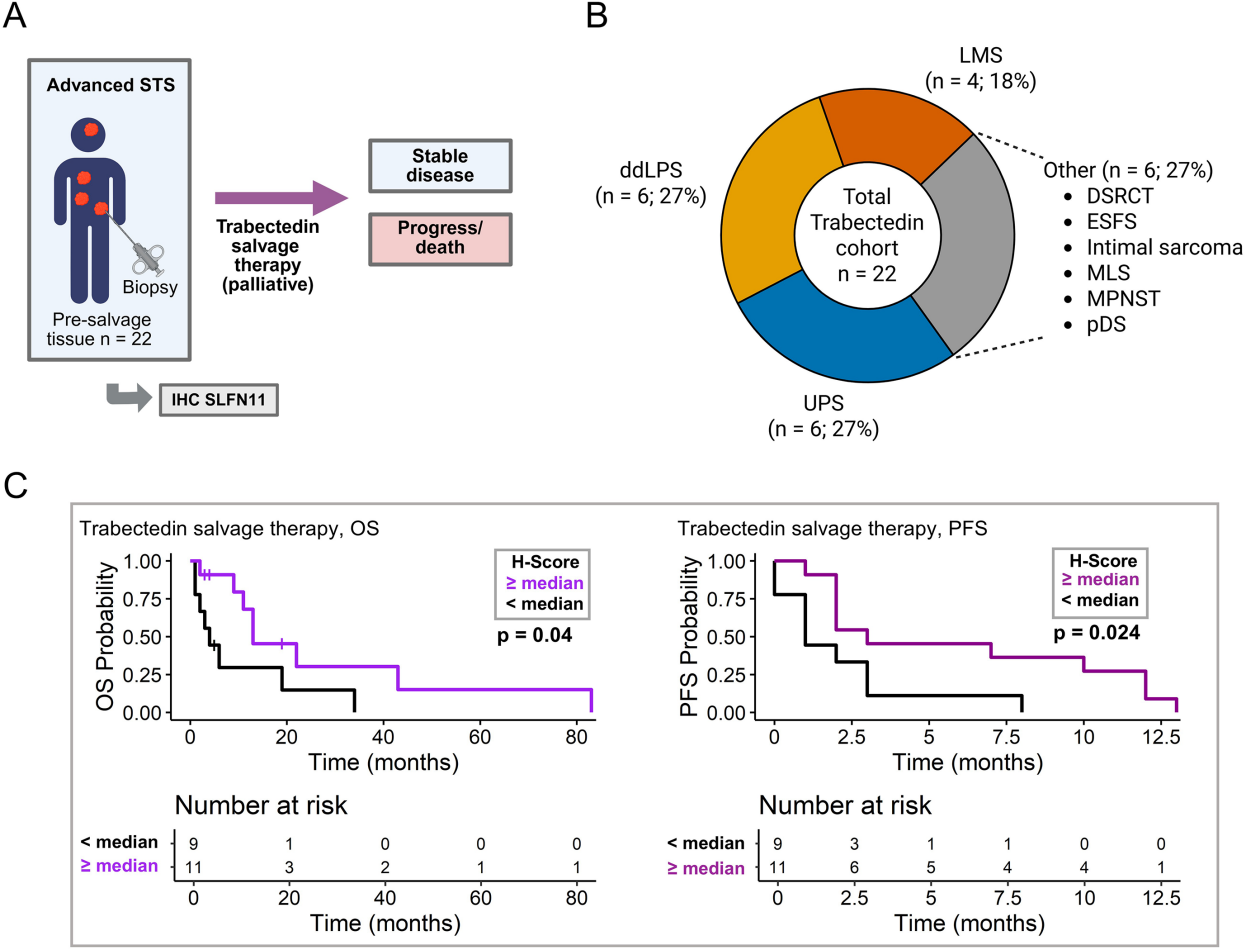
SLFN11 expression and survival in sub-cohort of patients treated with trabectedin. **(A)** Sub-cohort of patients with unresectable, relapsed, or metastatic STS receiving a palliative trabectedin salvage therapy. Created in BioRender. Simon, A. G. (2026) https://BioRender.com/vji640w. **(B)** Distribution of histological STS subtypes within the trabectedin sub-cohort. **(C)** Association of SLFN11 expression and overall survival (OS)/progression-free survival (PFS) in patients receiving palliative trabectedin. Survival differences were assessed using the log-rank test. Significant p values (p ≤ 0.05) are indicated in bold. IHC, immunohistochemistry; STS, soft tissue sarcomas; ddLPS, dedifferentiated liposarcoma; DSCRT, desmoplastic small round cell tumor; ESFS, epithelioid sclerosing fibrosarcoma; LMS, leiomyosarcoma; MLS, myxoid liposarcoma; MPNST, malignant peripheral nerve sheath tumor; pDS, pleomorphic dermal sarcoma; UPS, undifferentiated pleomorphic sarcoma.

In the last step, we performed exploratory SLFN11 expression assessment in a sub-cohort of n = 22 patients treated with trabectedin in a palliative salvage regimen (**Figure 5A**). The cohort included 6 cases of UPS, 6 cases of ddLPS, 5 cases of LMS, 6 more uncommon sarcoma subtypes (**Figure 5B**). All patients had surgically unresectable relapse and/or distant metastases, with lung, bones and brain being most common metastatic sites (**Supplementary Table 3**). In 16/22 (73%) patients, trabectedin was administered as second- or later-line systemic therapy following at least one prior chemotherapy. In three cases (14%), radiotherapy was administered before. In three cases (14%), trabectedin was administered without prior treatment (**Supplementary Table 3**). All patients had a progress eventually (median time to progress: 2.0 months, range 0–13 months), and 18/22 patients (82%) succumbed to their disease (median OS: 5.5 months, range 0–83 months). The latest available tissue of relapsed or metastatic tissue obtained prior to trabectedin treatment was assessed and SLFN11 expression was dichotomized by the global SLFN11 median (H-score and percentage score 2.5/2.5%). Patients with higher levels of SLFN11 showed a significantly longer OS than patients with low SLFN11 expression (13 vs. 4 months, log-rank p = 0.04) (**Figure 5C**) and a significantly longer PFS (3 vs. 1 months, p = 0.024) (**Figure 5C**). In univariate analyses, high SLFN11 expression showed a trend toward longer OS (HR 0.35, p = 0.054) and a significant association with longer PFS (HR 0.35, p = 0.038) (**Supplementary Table 11**). In multivariable analyses, including age at treatment start and tissue subtype (metastatic vs. non-metastatic), high SLFN11 levels showed a trend towards longer OS (HR 0.33, p = 0.053), but no association with PFS (HR 0.42, p = 0.127) (**Supplementary Table 12**).

## Discussion

While SLFN11 has recently emerged as a potential predictive biomarker for sensitivity to DNA-damaging agents in various cancers, its relevance in adult soft tissue sarcomas (STS) has not been investigated to date. Our study provides the first retrospective, real-world evidence that immunohistochemical (IHC) assessment of SLFN11 protein expression may represent a clinically meaningful biomarker in this heterogeneous disease group, including both common and less frequent STS entities (n = 242).

Since no standardized method for immunohistochemical SLFN11 assessment exists, we evaluated SLFN11 expression using both the H-score and the percentage score of SLFN11-positive tumor cells - two approaches commonly applied in recent retrospective studies. Gartrell et al. used the H-score in a cohort of 220 pediatric sarcomas, defining high or present SLFN11 IHC expression as a H-score > 0 (23). Several other retrospective studies have implemented the H-score across different cancers (17–19), with each study choosing different dichotomization for subsequent analyses. Kaczorowski et al. employed a four-tier system based on percentage of positive cells (any intensity) in the largest exploratory SLFN11 IHC analysis to date, covering more than 6600 samples across 127 tumor entities (28). Consistent with our results, they reported low SLFN11 expression in leiomyosarcomas (n = 76) and higher levels in dedifferentiated liposarcomas (n = 53). However, they frequently observed higher percentages of SLFN11-positive tumor cells, which may reflect differences in antibody selection, staining protocols, different subtype-specific cohort composition, or the potential inadvertent inclusion of SLFN11-positive immune cells - particularly in heavily inflamed, spindle-cell tumors. Takashima et al. assessed SLFN11 expression in another study including 700 tumors, also using a four-tier percentage-based system (29). Notably, they reported a substantial impact of immune-cell contamination on SLFN11 assessment in both mRNA and IHC analyses. Especially in highly inflamed tumors, SLFN11-positive immune infiltrates obscured true tumor-cell expression and could lead to falsely elevated SLFN11 estimates. To improve discrimination between spindle-shaped tumor cells and M2-like macrophages - the predominant immune-cell population in most sarcomas (30) - we performed additional staining for CD163, which subjectively facilitated accurate tumor-cell identification. Nevertheless, distinguishing tumor cells from immune cells might remain challenging even for experienced soft tissue pathologists, especially in more monotonous, low-grade STS, and supplemental markers may be required in highly inflamed tumors.

Importantly, SLFN11 expression was assessable with high interobserver concordance using both scoring methods, suggesting that - although a standardized SLFN11 scoring system and threshold for positivity are still lacking - both methods can be reliably applied in clinicopathological and research settings.

We observed a lower SLFN11 expression in post-treatment tissue after neoadjuvant chemotherapy, radiochemotherapy, and radiotherapy alone. In the chemotherapy-containing sub-groups, this reduction might reflect clonal selection, with elimination of SLFN11-high tumor cells sensitive to DNA-damaging agents and relative enrichment of resistant SLFN11-low clones. Differences in tumor composition after treatment, including fibrosis, immune infiltration, and sampling variation between biopsy and resection specimens, may additionally influence apparent expression changes.

Notably, a significant association between high SLFN11 expression and increased tumor regression was observed only in patients receiving chemotherapy-containing regimens. In contrast, as SLFN11 expression was not associated with response to radiotherapy alone, the decrease observed after radiotherapy might rather reflect an adaptive or epigenetic downregulation of SLFN11 than selective elimination of SLFN11-high clones. Several studies have described radiotherapy-induced upregulation of the chromatin regulator EZH2 in malignant and normal tissues as a resistance mechanism (31, 32). EZH2 can repress SLFN11 through H3K27me3 deposition at the SLFN11 locus, a process linked to treatment resistance in multiple experimental cancer models (33, 34). Despite limited sample sizes, our findings are consistent with the emerging role of SLFN11 as a key determinant of sensitivity to DNA-damaging chemotherapy, whereas radiotherapy efficacy in sarcomas might be governed by SLFN11-independent pathways.

Our findings in the cohort of primarily resected STS support this notion: High SLFN11 expression was an independent predictor of prolonged survival in patients receiving adjuvant chemotherapy or radiochemotherapy, but not radiotherapy alone. Similar associations between SLFN11 expression and improved response to DNA-damaging agents have been reported in several carcinomas, including small cell lung cancer, high-grade serous ovarian carcinoma, head and neck squamous cell carcinoma, and gastric cancer (17, 18, 20, 35). For sarcomas, however, retrospective data remain limited. Merlini et al. identified SLFN11 among eight genes associated with enhanced response to trabectedin and the PARP inhibitor olaparib in 32 adult bone and soft-tissue sarcomas using a Nanostring nCounter mRNA panel and RNA *in situ* hybridization (36). However, no clear association between SLFN11 expression and survival was observed, and IHC analyses were not performed. For IHC analyses, Federico et al. evaluated SLFN11 protein expression in a phase I study including Ewing sarcoma, desmoplastic small round cell tumors (DSRCT), rhabdomyosarcoma, and osteosarcoma, and observed that SLFN11-high tumors were more likely to respond to irinotecan and the PARP inhibitor talazoparib (22). Gartrell et al. reported high SLFN11 expression in 143 pediatric tumors, including neuroblastoma, Ewing sarcoma, osteosarcoma, and embryonal rhabdomyosarcoma (23). While SLFN11 expression in sarcoma cell lines was associated with increased sensitivity to SN-38 and talazoparib, no significant survival differences were observed in retrospective IHC analyses for patients with SLFN11-high tumors. This may reflect heterogeneous cohort composition, high subtype-dependent SLFN11 expression, and additional SLFN11-independent resistance mechanisms in heavily pre-treated tumors. Notably, 31% of the cohort consisted of translocation-associated sarcomas (Ewing sarcoma, alveolar rhabdomyosarcoma, DSRCT) for which distinct biological behavior with partially stem-like, SLFN11-independent resistance mechanisms to chemotherapeutics have been described (1, 37–39). Complementing the clinical observations, various previous mechanistic studies in sarcoma cell lines have demonstrated that SLFN11 expression enhances sensitivity of sarcoma cell lines to several DNA-damaging agents, including ATR inhibitors and lurbinectedin (40), trabectedin (41), and irinotecan (42), supporting a causal link between SLFN11 and chemosensitivity.

In advanced STS, SLFN11 remained a prognostic marker of improved outcomes. High SLFN11 expression was associated with longer survival in the sub-cohorts treated with palliative first-line chemotherapy or second- and later-line trabectedin. To date, chemotherapy regimens for advanced or metastatic sarcomas achieve only modest response rates of approximately 10 - 35% (5, 7, 8). These regimens are commonly associated with substantial grade toxicities, including neutropenia, anemia, and febrile neutropenia (5, 8, 9). Consequently, there is an urgent need to identify patients most likely to benefit from palliative therapy. Our findings suggest that SLFN11 assessment may support treatment selection and help guide the intensity of palliative therapy in routine clinical practice.

We observed intra-patient variation in SLFN11 expression across matched primary, recurrent, and metastatic samples, suggesting that SLFN11 is not static over the disease course. Consistent with these findings, Winkler et al. assessed SLFN11 expression in a breast cancer cohort and reported higher SLFN11 expression in metastatic tissue samples (43). This emphasizes the potential value and necessity of a re-biopsy prior to initiating systemic therapy, particularly when prior treatments or long disease intervals may have altered tumor biology.

While this study comprises the largest real-world cohort of adult STS to date to assess the potential of SLFN11 as a biomarker, several limitations must be acknowledged. As in most retrospective single-center sarcoma studies, relatively small sub-cohort sizes and both treatment and histological heterogeneity may limit statistical power and introduce selection bias. Moreover, the inherent difficulty of capturing spatial SLFN11 expression in heavily inflamed STS - particularly when relying on TMA cores - was evident even for experienced pathologists, though we partially addressed this through supplementary immunohistochemistry for intra-tumoral macrophages and whole-slide sections. In addition, the global SLFN11 median used for dichotomization in this study, although yielding consistent statistical results, represents only one of several potential thresholds. SLFN11 IHC is not yet standardized, and assessment results may vary depending on antibody selection, staining platform, and scoring method. Thus, large multicenter studies and broader datasets will be required to establish clinically meaningful and reproducible cutoffs for treatment decision-making.

## Conclusion

This study provides the first comprehensive analysis of immunohistochemical SLFN11 expression as a prognostic and potentially predictive biomarker in adult soft tissue sarcomas. High SLFN11 expression was consistently associated with greater tumor regression and improved survival across multiple chemotherapy-related treatment settings. While prospective validation and standardized assessment are still needed, these findings highlight the potential clinical utility of SLFN11 to inform treatment decisions in STS.

## Data Availability

The original contributions presented in the study are included in the article/**Supplementary Material**. Further inquiries can be directed to the corresponding author/s.
